# Percutaneous Vocal Fold Lateralization in children — a case series of a Brazilian tertiary pediatric hospital (pediatric vocal fold lateralization cases in a Brazilian hospital)

**DOI:** 10.1016/j.bjorl.2024.101469

**Published:** 2024-07-14

**Authors:** Bárbara Duarte Salgueiro, Neemias Santos Carneiro, Hemiliy Izabel Alves Neves, Isabel Saorin Conte, Rita Carolina Pozzer Krumenauer Padoin, Renata Loss Drummond, Marcelo Neves Lubianca, José Faibes Lubianca Neto

**Affiliations:** a Programa de Fellowship do Serviço de Otorrinolaringologia (ORL) Pediátrica do Hospital da Criança Santo Antônio (HCSA)/ Universidade Federal de Ciências da Saúde de Porto Alegre (UFCSPA); b Serviço de ORL Pediátrica do HCSA e Serviço de ORL da ISCMPA/HCSA/UFCSPA; c Programa de Residência Médica do Serviço de ORL da Irmandade Santa Casa de Misericórdia de Porto Alegre (ISCMPA); d Pontifícia Universidade Católica do Rio Grande do Sul (PUCRS); e Serviço de ORL Pediátrica do HCSA, Serviço de ORL da ISCMPA, Programa de Pós-Graduação em Pediatra da UFCSPA

**Keywords:** Bilateral vocal cord paresis, Vocal fold, Percutaneous lateralization, Tracheostomy, Vocal fold paralysis

## Abstract

**Objectives:**

Percutaneous Vocal Fold Lateralization (PVFL) consists of external fixation with non-absorbable percutaneous suture of the vocal fold in a lateral position, under direct glottic visualization. The objective of this study is to determine the effectiveness of PVFL in a university pediatric hospital, as well as to describe the potential risks and complications of the surgery.

**Methods:**

Retrospective cohort study, with data collected from electronic medical records. The study was approved by the Research Ethics Committee of the institution. Surgeries were performed with the modified Lichentenberg technique and data, and outcomes were analyzed.

**Results:**

Six patients with Bilateral Vocal Fold Paralysis who underwent Percutaneous Vocal Fold Lateralization were evaluated. Three patients were male. The age at diagnosis ranged from 2 to 132 days (mean 10.5 days). The reason for investigating the upper airway was the presence of increased work of breathing and stridor. Five patients had a favorable clinical evolution, with spontaneous ventilation in room air and absence of stridor or ventilatory effort, without the need for tracheostomy. Surgical results in this series corroborate the findings of other similar cohorts, which showed Percutaneous Vocal Fold Lateralization as a safe and effective procedure in avoiding tracheostomy or allowing decannulation in children with Bilateral Vocal Fold Paralysis.

**Conclusions:**

PVFL seems to be a safe and effective procedure, but it has morbidity, due to immediate, and probably late, non-serious complications. Studies with a larger number of patients, with longer follow-up and using a controlled and randomized clinical design are needed to establish the role of PVFL in the treatment of BVFP in newborns and infants.

**Levels of evidence:**

Level 4 (step 4).

## Highlights

• Surgical results showed PVFL as a safe and effective procedure.
• PVFL may avoid tracheostomy and allow decannulation in children with BVFP.
• PVFL is promising, as the tracheostomy has significant morbidity.
• Five of six patients had a favorable clinical evolution, after PVFL.
• In our series of cases, there were no cases of reversal of vocal fold lateralization.

## Introduction

Bilateral Vocal Fold Paralysis (BVFP) in children is a rare condition, with an estimated incidence of 0.75 cases/1,000,000 million births/year. It is the 2nd most common congenital cause of stridor in newborns, responsible for about 12.9% of the cases of stridor.^1^, ^2^ Usually, the child presents with inspiratory stridor and signs of acute respiratory distress, but crying is normal. Although the intensity of respiratory distress varies, there may be acute respiratory failure and demand for non-invasive ventilation, intubation, or even urgent tracheostomy.^3^ The most common causes of BVFP in children are neurological (26%–56% — Arnold–Chiari malformation, myelomeningocele, hydrocephalus), traumatic (20% — endotracheal intubation and birth trauma), iatrogenic (associated with cardiothoracic surgeries) and idiopathic (41%–53%).^1^, ^4^

The rate of spontaneous recovery of vocal fold mobility varies widely among series and is correlated with the underlying etiologic process. Those neurological and idiopathic cases have a greater chance of recovery (around 50%–77%).^5^, ^6^, ^7^, ^8^, ^9^ The reason for the consensus to avoid definitive procedures on the laryngeal framework before 2 years of age is based is this high rate of spontaneous recovery of the vocal fold motion.^10^

Tracheostomy is still considered the surgical treatment of choice, but it has a major impact on children’s quality of life and a higher rate of complications than in adults.^11^ In addition, it entails a social and financial burden on the family and the health system. Therefore, less invasive surgical options are welcome, especially if they have some degree of reversibility, considering the possibility of spontaneous recovery of vocal fold mobility.

Percutaneous Vocal Fold Lateralization (PVFL) consists of external fixation with non-absorbable percutaneous suture of one of the vocal folds in a lateral position, under direct endoscopic or microscopic visualization.^12^, ^13^, ^14^, ^15^ It is a simple technique, potentially reversible and with low associated morbidity, which may avoid tracheostomy, or even enable decannulation. There are case series showing efficacy (avoidance of tracheostomy) close to 100%.^16^

The aim of this study is to determine the effectiveness of PVFL in a university pediatric hospital, as well as to describe the potential risks and complications of the surgery.

## Methods

A retrospective cohort study was performed, with data collected from electronic medical records. The study was approved by the Research Ethics Committee of the Hospital of the Santa Casa Hospital Complex in Porto Alegre (Opinion 5.695.661).

### Patient selection and data collection

All pediatric patients with BVFP who underwent PVFL at the Santo Antônio Children’s Hospital of the Santa Casa Hospital Complex in Porto Alegre were included. The procedures were performed between March 2020 and November 2022.

### Surgical technique

**Figure 1:**
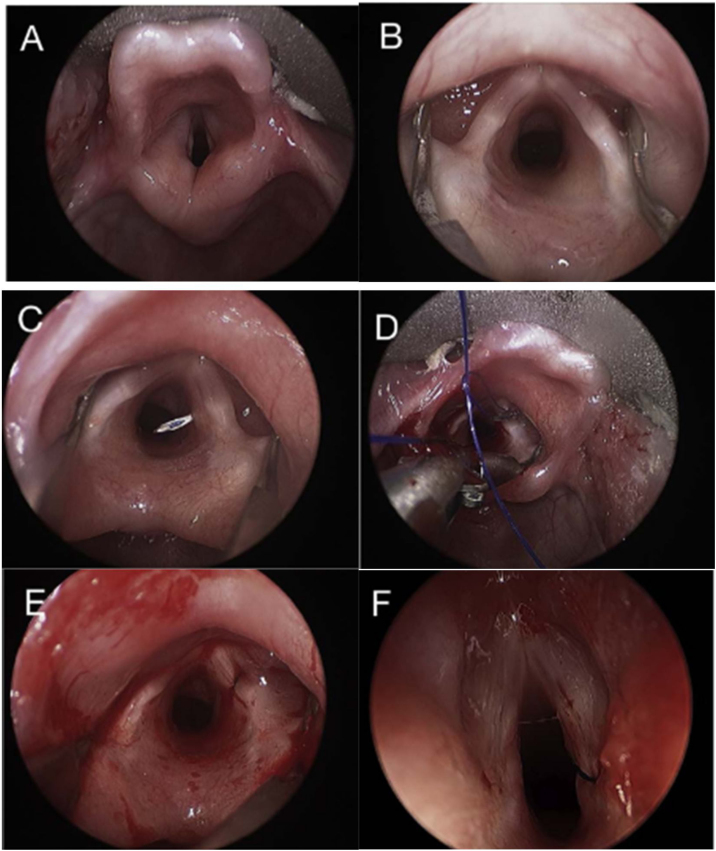
Rigid endoscopy photographs demonstrating in sequence from the beginning to the end of the surgery (A–F).^16^

**Figure 2:**
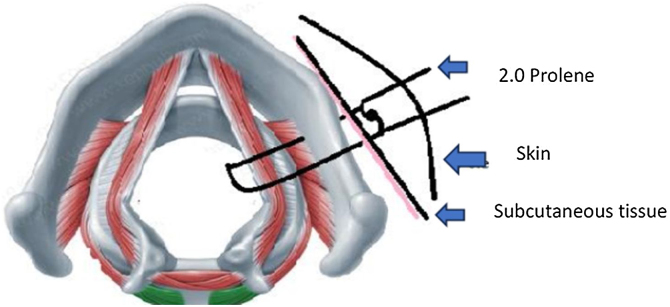
Schematic illustration of the technique of percutaneous suturing and lateralization of the right vocal fold.

A modification of the original technique described by Lichtenberger and Toohill^17^ for PVFL was used. Prior to the procedure, Bilateral Vocal Fold Paralysis was confirmed through flexible nasofibrolaryngoscopy with the patient awake in the operating room. All patients underwent suspension direct laryngoscopy using the Lindholm pediatric laryngoscope and a zero-degree 4 mm sinuscope while spontaneously breathing under general anesthesia. We also use the vocal fold retractor to improve visualization in all procedures. The surgery begins with a 4 mm neck incision made in a relaxed skin tension line at inferior border of thyroid cartilage, 1 cm lateral to midline. The method of laterofixation is based on inserting a needle (18-gauge intravenous catheter — Abocath™) through the skin incision and thyroid cartilage exactly under the vocal cord so that the end of the needle is located in the lumen of larynx. A 3–0 prolene surgical thread (suture #2) was loaded in the placed needle into the larynx and pulled up to the mouth with a micro laryngeal forceps, keeping the opposed end of the thread pulled away from the neck. The identical needle is inserted above the first one in such a way that the end of the needle is placed above the vocal cord. Then a 4.0 prolene suture loop (suture #3) was loaded in the needle and the loop was pulled up to the mouth in the same way as suture #1. After the end of suture #1 is passed through the loop of suture #2, the suture loop was then pulled out of the back of the needle, bringing suture #1 with it. While observing the lateralization of vocal cord under direct visualization, suture #1 was tied over deep in the subcutaneous tissue. The skin of the lateral neck is closed with 4.0 mononylon thread. Endoscopic photographs of the various steps of the technique are show in Fig. 1, and schematic illustration of the BVFL is shown in Fig. 2.

### Outcomes and data analysis

The primary outcome was defined as the resolution of glottic insufficiency (manifested as stridor, intercostal, subcostal, and sternal retractions) avoiding tracheostomy or, in cases already tracheostomized, allowing decannulation. Secondary outcomes were occurrence of trans or postoperative complications, dysphonia, and dysphagia. The impacts on voice were evaluated through the subjective perception of the caregivers and examiners team, while swallowing was evaluated through the clinical observation and diet progression.

## Results

### Demographic and clinical characteristics

Six patients with BVFP who underwent PVFL were evaluated (Table 1). Three patients were male. The age at diagnosis ranged from 2 to 132 days (mean 10.5 days). The reason for investigating the upper airway was the presence of increased work of breathing and stridor. Prior to the procedure, two patients did not need ventilatory support but, due to comorbid conditions, presented respiratory distress during episodes of oral feeding, as well as episodes of sporadic apneas during sleep. They did not present, however, severe desaturations. Another three patients needed supplemental oxygen due to respiratory distress that also hindered adequate oral feeding. In these cases, however, the indication for ventilatory support was also linked to their comorbidities. Only one patient had already been tracheostomized before BVFL due to previous unsuccessful extubating attempts associated to complex cardiac comorbidities. Among the comorbidities, 3 patients presented heart disease and 1 patient had a giant arachnoid cyst. Two patients had vocal fold paralysis secondary to previous cardiac surgery, one had neurological etiology, and 3 were idiopathic.

**Table 1:** Clinical and demographic characteristics of the sample.

	Sex	Age at diagnosis (days)	Age at surgery (days)	Comorbidities	Synchronic lesion in the airway	Ventilatory support (preop)	Etiology
1	M	5	10	No	No	NC	Idiopathic
2	F	132	149	Corrected PDA, prematurity (29-weeks), seizures	Laryngomalacia type I + II	NC	Iatrogenic (cardiac surgery)
3	F	2	21	No	No	NC	Idiopathic
4	M	29	605	PDA, IAC, PAS, left pulmonar artery hypoplasia, brain venous thrombosis, IA	Tracheal stenosis with RMB stenosis	Trach (RA)	Iatrogenic (cardiothoracic surgery)
5	M	2	9	Giant arachnoid cyst in posterior fossa	No	RA	Neurological
6	F	16	23	PDA, PFO, AoC, MR, BAV	Post extubation laryngitis	RA	Iatrogenic (cardiothoracic surgery)
	Averages (SD)	10.5 (50.57)	22 (235.85)				

PDA, patent ductus arteriosus; PAS, pulmonary artery sling; IAC, interatrial communication; NC, nasal cannula; Trach, tracheostomy; RA, room air; PFO, patent foramen oval; IA, imperforate annus; SD, standard deviation; AoC, aortic coarctation; MR, mitral regurgitation; BAV, bicuspid aortic valve; RMB, right main bronchus; Preop, preoperative.

In relation to the presence of synchronous airway lesions at the time of diagnosis, one patient had mixed laryngomalacia (types I + II), which was corrected by concomitant supraglottoplasty.

### Intraoperative and immediate postoperative period

The surgical time ranged from 71 to 115 min. There were no intraoperative complications. The first two patients who underwent PVFL in this case series were intubated in the immediate postoperative period, due to moderate respiratory disfunction with desaturation. The other patients remain stable after surgery. They were maintained on supplemental oxygen through a nasal catheter with good tolerance and were progressively weaned from oxygen during the first postoperative day. Patients #1, 2, 3 remained with a nasoenteral tube for 5 days and then returned to exclusive oral diet. One patient (#6) remained with mix diet for 127 days, date of the last revision. Data related to the procedure and its outcomes are shown in Table 2.

**Table 2:** Data and outcomes of the procedures.

	Surgery		Time (POETI)	Ventilatory support after surgery	Dysphagia		Follow up (days)
	VF	Time (min)			Immediate postoperative	aPO (days after surgery)	
1	LVF	115	1	48 h	Yes — NETF	5 days	932
2	LVF	89	1	48 h	Yes — NETF	5 days	671
3	RVF	93	0	6 h^c^	Yes — NETF	5 days	229
4	LVF	75	0	bTrach after surgery	Yes — NETF	5 days	250
5	LVF	99	0	6 h^c^	Yes — NETF	5 days	48
6	LVF	71	0	6 h^c^	Yes — NETF	Still on mixed diet	127
Average (SD)			90.3 (20.4)				
						Average (SD)	239.5 (347.3)

	Associated procedures	Post-operative complications	Revision procedures	Recovery of mobility
1	No	Surgical wound infection and granuloma (19° POD) VF granuloma (22° and 43° POD)	Granuloma removal VF (22° and 43° POD)	No
2	Supraglottoplasty	Granuloma VF (7° POD)	Granuloma removal VF	Partial (RVF)
3	No	Suture dehiscence	RVF (21° POD)	No
4	No	Suture dehiscence	LVF lateralization (63° POD)	Partial (RVF)
5	No	No	No	No
6	No	No	No	No

LVF, left vocal fold; RVD, right vocal fold; POETI, post operative endotracheal intubation; ETI-PO, endotracheal intubation post operative; FU, follow up; Trach, tracheostomy; AA, ambient air; p.o, per oral; NETF, nasoenteral tube feeding; VF, vocal fold; SD, standard deviation; POD, postoperative day.

a Indicates the days until complete oral feeding after oral rehabilitation.

b It was impossible to remove the tracheostomy in the post-op period, and the tracheostomy was kept on ventilation without the need for Ayre.

c Time to remove nasal catheter with supplemental oxygen.

### Main outcome

Table 2 summarizes data related to functional outcomes. Regarding the main outcome, five patients had a favorable clinical evolution, with spontaneous ventilation with no desaturations or stridor, not requiring tracheostomy. Patient #4 was already wearing a tracheostomy prior to diagnosis due to the concomitance of synchronous lower airway lesions and to surgery for cardiac comorbidities. The decannulation attempt failed late in this patient, requiring tracheostomy maintenance to the date.

The patients remained in the hospital for 15–20 days, and all of them were reassessed in the operating room at least 7 days after the initial surgical procedure. Close monitoring was required, with the need for additional interventions in 4 cases. Time to hospital discharge varied among patients and it was considered just when breathing and feeding were judged adequate, with frequent and rigorous reassessments before the discharge.

### Secondary outcomes

In the postoperative period, all patients received a nasogastric tube, with progression to an oral diet according to the clinical evaluation of the medical staff and the swallowing therapist. At the time of the last assessment, all patients were safely fed orally, 5 of them exclusively by mouth. Patient #6 is still on a mixed diet (oral on demand, with nutritional supplement via nasogastric tube). Subjectively, moderate dysphonia was observed with hoarse and weak crying in all patients. No swallowing assessment instrument was applied.

### Complications

Two patients underwent surgical reinterventions due to suture dehiscence. Two patients had small non-obstructive peri-suture vocal fold granulomas that were resected endoscopically in revisional reinterventions. One patient had granuloma formation on the skin and wound infection, which was treated with resection of skin granuloma and oral antibiotics.

### Clinical follow-up and functional recovery

The follow-up time ranged from 48 to 932 days. These patients periodically returned for revisional evaluations, undergoing airway endoscopic examinations in the operating room. Until the last evaluation, no patient had completely recovered the movement of the vocal folds, but in two of them there was partial recovery of the movement of the contralateral vocal fold. One patient remains tracheostomized due to synchronous lesion of the lower airway (case #4) and one has mild stridor on exertion (case #6). The immediate and long-term results of the procedure can be seen in Table 2.

## Discussion

Surgical results in this case series corroborate the findings of other similar cohorts, which showed PVFL as a safe and effective procedure that can avoid tracheostomy or allow decannulation in children with BVFP.^16^, ^17^, ^18^, ^19^, ^20^ However, the potential although not serious complications and some doubts about reversibility should influence the choice of PVFL over other techniques.

Some authors pointed out as a negative aspect of PVFL the need for revisional or adjunct procedures.^21^ In our series, patients #1 and #2 needed revisional endoscopic procedures to remove granulation tissue, while patients #3 and #4 needed new lateralization procedures due to suture dehiscence. Granulomas are possible complications in the postoperative period, especially in regions that undergo excessive manipulation with secondary laceration and edema. In the case of vocal fold lateralization, the material of the suture thread used, as well as the need for surgical revisions with additional manipulations (endoscopic exams) and/or surgical site infection, all can be included in the reasons for the occurrence of granulation tissue in the current study.

None of the patients in the series had full recovery of vocal fold mobility. Two patients, however, had partial recovery of the mobility of contralateral vocal fold. The low rate of vocal fold mobility recovery is difficult to explain. Perhaps the short duration of our follow-up could have been insufficient to evaluate this outcome. Also, the intervention itself might have influenced the “spontaneous recovery” because there is a potential for ankylosis or fixation of the cricoarytenoid joint. The learning curve may also have been the cause as it is associated with excessive manipulation. This suspicion increases when it is realized that only the first two cases required intubation in the postoperative period, probably due to hypermanipulation during surgery. Finally, perhaps the use of the Rovo’s needle system,^22^ with more controlled insertions of the needle, could obviate some of the factors listed above.

We use a nasogastric tube in the immediate postoperative period 6 patients. All underwent follow-up with swallowing therapist, managing to progress to an exclusive oral diet, but patient #6 is still on a mixed diet (oral and via nasogastric tube). Mathur et al.^20^ demonstrated the occurrence of transient aspiration in 2 of 10 patients in their series, while Tan et al.^18^ described that all patients submitted to PVFL presented transient aspiration on videoendoscopic evaluation of swallowing in the first postoperative month.

Objective voice assessment was limited by the patients’ age at the time of the procedure, as well as by the lack of a standard instrumental voice assessment for this age group. Although we do not have data from objective scales of voice assessment, the subjective perception of the team and caregivers ranked the voice as acceptable at the time of the final assessment. There is little in the literature about the impacts of this procedure on phonation in the pediatric population, but Rovó et al.^22^ demonstrated (through aerodynamic studies, graphic analysis and voice questionnaires) an acceptable vocal quality in adults, even in patients who do not present any degree of mobility recovering. The same group of authors demonstrated, in a cadaveric study, a more favorable posterior glottic configuration with external lateralization in relation to the classic latero-fixation of the vocal fold, transverse cordotomy or arytenoidectomy.^23^ However, there is still no standardization regarding the use of existing scales for vocal assessment in the postoperative period in this age group.^24^ More studies are necessary in the future to quantitatively compare voice quality among different techniques for Bilateral Vocal Fold Paralysis in children. They should use acoustic data obtained through the lab voice apparatus, and data from b-VHI and from pediatric voice-related quality of life questionnaires.

The reversibility of the procedure is not that clear in the literature. Some claims for reversibility in 75% of cases. In our series it was chosen to maintain the suture even in those patients who presented some degree of functional recovery, due to their good ventilatory adaptation, as well as the satisfaction of the parents regarding the voice and swallowing; it is important to report that the follow-up of our patients is still ongoing, and none of them has yet entered puberty, which represents a complex moment of maturation of larynx. There is no established age for removing the suture, or even if it is necessary. Montague et al.^16^ describe suture removal in 3 cases (caused by insufficient lateralization, aspiration, and dysphonia, respectively). In cases of removal due to complications, there was an improvement in the symptoms of dysphagia and dysphonia, without respiratory worsening, suggesting some degree of tension even after suture removal. The author also discussed what happens to the suture in the long term and postulates that there is some degree of submucosal cordotomy, followed by re-epithelialization over the suture, since the thread does not remain visible in subsequent endoscopic evaluations. Montague et al.^16^ also questioned the existence of some degree of ankylosis or fixation of the cricoarytenoid joint.

In the 6 cases studied, frequent reassessments were necessary in the first postoperative weeks. The granulomas, even they were small, were excised early because of the potential for growth, fibrosis, and obstruction. Also, two cases required subsequent re-lateralization, because of disappearance of prolene thread and returning of vocal fold to the original position. Because of these early complications, we recommend that the first endoscopic reassessment should not be postpone beyond 14 days. Episodes of gagging during feeding are not rare in the first days, and we suggest maintenance of the nasogastric tube at least for five days and evaluation and treatment with a swallowing therapist as soon as possible in the postoperative period. Discharge from the hospital should be delayed until the patient is stable in terms of ventilation, ideally with no requirements of supplemental oxygen and when safe return for exclusive oral intake or at least for mixed diet are achieved.

### Limitations

This retrospective case series has limitations such as small sample and absence of a control group. The choice of surgical procedure was made prior to the study and was made by the joint decision of the surgeon and the family, representing a selection bias, since in Brazil parents generally do not accept tracheostomy initially and try to avoid it at all costs. Being a unicentric study, the outcomes may have been influenced by the clinical and demographic profiles of the patients. The short follow-up time limits the understanding of the long-term impacts of PVFL. The results are also restricted by the lack of objective assessment of swallowing and voice through standardized methods.

## Conclusions

PVFL seems to be a safe and effective procedure, but it has morbidity due to immediate and late, non-serious, complications. It seems promising, however, since the alternative procedure, tracheostomy, although also reversible, has significant morbidity and mortality, in addition to all inherent limitations to patient and caregivers. Studies with larger number of patients, with longer follow-up and using a controlled and randomized design are needed to establish the role of PVFL in the treatment of BVFP in newborns and infants.

## Authors’ contributions

Jose Faibes Lubianca Neto; Rita Carolina Pozzer Krumenauer Padoin; Renata Loss Drummond: Methodology, Conceptualization, Supervision, Project Administration. Barbara Salgueiro Duarte: Writing-Original Draft; Neemias Santos Carneiro: Writing-Review & Editing; Visualization; Marcelo Lubianca: Methodology, Conceptualization; Isabel Saorin: Visualization; Hemily Neves: Visualization.

## Funding

This research did not receive any specific funding from public funding agencies, commercial or not-for-profit organizations.

## Conflicts of interest

The author declares no conflicts of interest.
